# 168. Closing Time: A Quasi-experiment Comparing Time to Optimal Therapy using Traditional Identification and Susceptibility Methods, Rapid Identification, or Rapid Identification with Phenotype for Gram-negative Bloodstream Infections

**DOI:** 10.1093/ofid/ofac492.246

**Published:** 2022-12-15

**Authors:** Rachel Perry, Nicholas J Mercuro, Nicholas J Mercuro, Kristina Rokas Connolly, Eliza W Dollard, Patricia Stogsdill, Minkey Wungwattana

**Affiliations:** Maine Medical Center, Charlottesville, Virginia; Maine Medical Center, Charlottesville, Virginia; Maine Medical Center, Charlottesville, Virginia; Maine Medical Center, Charlottesville, Virginia; Maine Medical Center, Charlottesville, Virginia; Maine Medical Center, Charlottesville, Virginia; Maine Medical Center, Charlottesville, Virginia

## Abstract

**Background:**

Bloodstream infections (BSI) are a major cause of morbidity and mortality. Compared to traditional microbiology methods, rapid diagnostic testing (RDT) provides prompt organism identification and antibiotic susceptibility results. This leads to earlier opportunities to optimize therapy, which may improve patient outcomes.

**Methods:**

This was a single center quasi-experimental study of hospitalized adults with Gram-negative (GN) BSI, comparing the management and outcomes across three periods: pre-RDT (traditional identification and susceptibilities with BD Phoenix®, December 2014 to September 2016), RDT1 (Nanosphere Verigene®, December 2016 to December 2019), and RDT2 (Accelerate Pheno™, February 2020 to September 2021). The primary outcome was time to optimal therapy (TTOT). Secondary outcomes included time to oral antibiotic step-down, hospital length of stay (LOS), incidence of *Clostridioides difficile* infection, and 30-day inpatient mortality. Chi-squared and Kruskal-Wallis tests were used to compare categorical and continuous variables. Significance values for the primary endpoint were adjusted with Bonferroni correction to account for multiple pairwise comparisons.

**Results:**

Of 296 included patients, 100 were in the pre-RDT, 98 in the RDT1, and 98 in the RDT2 arms. The most common organisms were *Escherichia coli* (64.5%) and *Klebsiella* species (20.3%). The TTOT (median, interquartile range) in the pre-RDT, RDT1, and RDT2 groups were 46 (7-61), 30 (0-53), and 12 (0-28) hours (P< 0.001), respectively. The time to oral antibiotic step-down and hospital LOS was similar between groups. There was no difference in *C. difficile* infection or 30-day inpatient mortality (Table 1).

**Table 1.**
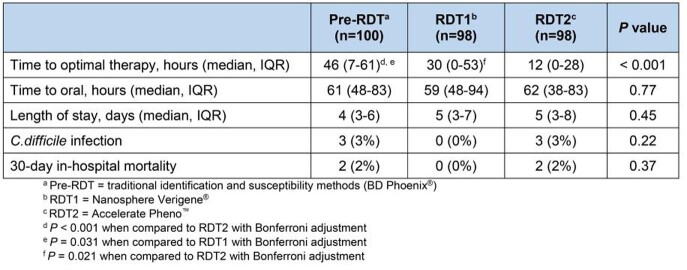
Comparison of Outcomes

**Conclusion:**

In patients with GN BSI the TTOT in RDT1 was shorter when compared to traditional susceptibility methods. Compared to both rapid identification alone and traditional identification and susceptibility methods, RDT2 improved time to optimal therapy. Larger, controlled studies are needed to examine the clinical impact of different RDT methods for Gram negative bloodstream infections.

**Disclosures:**

**Rachel Perry, PharmD**, Accelerate Diagnostics: Investigator initiated funding for research presentation; no funding for study design or data analysis **Minkey Wungwattana, PharmD, BCIDP**, Accelerate Diagnostics: Investigator initiated funding for research presentation; no funding for study design or data analysis.

